# DNA analysis of molluscs from a museum wet collection: a comparison of different extraction methods

**DOI:** 10.1186/s13104-016-2147-7

**Published:** 2016-07-18

**Authors:** Katharina Jaksch, Anita Eschner, Thomas V. Rintelen, Elisabeth Haring

**Affiliations:** Central Research Laboratories, Natural History Museum Vienna, Burgring 7, 1010 Vienna, Austria; Department of Integrative Zoology, University of Vienna, Althanstraße 14, 1090 Vienna, Austria; Third Zoological Department, Natural History Museum Vienna, Burgring 7, 1010 Vienna, Austria; Leibniz Institute for Research on Evolution and Biodiversity, Museum für Naturkunde, Invalidenstraße 43, 10115 Berlin, Germany

**Keywords:** Museum material, Ethanol preserved specimens, Mucopolysaccharides, Molluscs, Ancient DNA, DNA extraction methods

## Abstract

**Background:**

DNA isolation and PCR amplification from molluscan taxa is considered as problematic because polysaccharides in tissue and mucus presumably co-precipitate with the DNA and inhibit the activity of DNA polymerase. In the present study we tested two common extraction methods on specimens from the mollusc collection of the Natural History Museum Vienna (NHMW). We analysed a broad variety of taxa covering a large temporal span (acquisition years 1877 to 1999), which distinguishes our study from previous ones where mostly fresh material was used. We also took other factors into account: effects of sample age, effects of formaldehyde treatment and taxon-specific problems. We used several primer combinations to amplify amplicons of different lengths of two mitochondrial genes: *cytochrome c oxidase subunit 1* (*COI*) and *16S rRNA* gene (*16S*).

**Results:**

Overall PCR success was 43 % in the 576 extractions (including all primer combinations). The smallest amplicon (~240 bp) showed the best results (49 % positive reactions), followed by the 400 bp amplicon (40.5 %). Both short sections yielded significantly better results than the 700 bp long amplicon (27 %). Comparatively, the Gen-ial-First, All-tissue DNA-Kit—extraction method performed significantly better than Promega-Tissue and Hair Extraction Kit. Generally, PCR success is age-dependent. Nonetheless, we were able to obtain the longest amplicon even from 137-year-old material. Importantly, formaldehyde traces did not totally inhibit amplification success, although very high concentrations did.

**Conclusions:**

Museum material has gained importance for DNA analysis in recent years, especially for DNA barcoding projects. In some cases, however, the amplification of the standard barcoding region (partial sequence of the *COI*) is problematic with old material. Our study clearly shows that the *COI* barcoding region could be amplified in up to 49 % of PCRs (varying with amplicon length), which is, for museum samples, quite a high percentage. The difference between extraction methods was minimal and we recommend using an established kit for a first attempt because experience and routine in handling might be more important than slight performance differences of the various kits. Finally, we identify fixation, storage, sample conservation and documentation of the specimens’ history rather than the DNA extraction method to be the most crucial factors for PCR success.

**Electronic supplementary material:**

The online version of this article (doi:10.1186/s13104-016-2147-7) contains supplementary material, which is available to authorized users.

## Background

Although genomic DNA extraction has been a basic standard procedure in molecular biology since the 1980s [1] and numerous protocols are in use (most of them commercial), DNA isolation and subsequent PCR amplification from mollusc taxa remain problematic. Polysaccharides present in the mucus and tissues are considered as a main problem: they probably co-precipitate with the DNA and inhibit the activity of DNA polymerase [2–5]. A strong inhibitory effect of polysaccharides of a myxomycete on DNA polymerase activity was found in an investigation of Shioda and Murakami-Murofushi [6], suggesting that the presumed effects of mucopolysaccharides of molluscs might be similar. Several studies have introduced (e.g. [4, 7]) or compared (e.g. [3, 8]) DNA extraction protocols in molluscs to optimize PCR success. Most, however, were performed with relatively fresh material. Molecular phylogenetic analyses on Alpine land snails performed in our lab (e.g. [9–14]) suggested that several extraction methods (as indicated by PCR success) perform equally well, although these observations were not analysed systematically. Another factor potentially influencing the PCR success in molluscs, the drowning method, was investigated by Kruckenhauser et al. [15]. The traditional procedure to kill snails for preservation is drowning in water for 12–48 h to obtain well-relaxed soft bodies. This method was criticized by Schander and Hagnell [16] and is suspected to reduce the possibility of obtaining DNA suitable for PCR. Nonetheless, the procedure of Kruckenhauser et al. [15] proved to deliver suitable material for anatomical, morphological as well as DNA-based methods. Recently, a new microwave-based method has been published by Galindo et al. [17] to prepare mollusc tissue for DNA extraction as well as anatomical studies. Besides the above-mentioned approaches to investigate DNA quality in molluscs, which were based on comparatively fresh samples, no comprehensive investigation of old specimens stored in museum collections has been performed so far. The present study was designed to test two extraction methods on a high number of specimens from the mollusc collection of the Natural History Museum Vienna (NHMW). We analysed a broad variety of mollusc taxa and covered a large temporal span regarding sample age. Beyond comparing the two commonly used extraction methods, we took other factors into account: age effects, effects of formaldehyde treatment, and taxon-specific differences in results.

## Methods

The present study was initiated by the third author (TvR) in the course of the SYNTHESYS 2, Joint research activity 5, which addressed the question how the “mucopolysaccharide problem” affects DNA analyses of biological samples after prolonged storage in ethanol. We analysed ethanol preserved specimens from a total of 72 glass jars of the NHMW mollusc wet collection which dates back to more than 300 years. Concentration of ethanol used in the collection is ~75 % (jars are controlled regularly and replenished with 75 % ethanol [18]). Two individuals were randomly chosen from each jar (i.e., 144 individuals altogether; Table 1). Two tissue samples (max. 1 mm^3^) were taken from each individual, each of which was divided and processed with two different extraction methods. Three of the taxa selected are common species of which enough material is available in many museum collections: *Cepaea nemoralis* (20 individuals investigated), *Dreissena polymorpha* (32 individuals), and *Viviparus acerosus* (12 individuals). This allows a future comparison of results with these species between several museums. Beside these three species, we aimed to select a broad variety of taxa of the phylum Mollusca. Therefore, we chose 17 additional taxa of four different classes, with a strong focus on land gastropods (9 taxa). The collection dates of the chosen samples ranged from the years 1877 to 1999 (according to the labels). In general all jars of the NHMW mollusc wet collection are filled with 75 % ethanol and are regularly checked and filled up. Nonetheless, we don’t know the detailed treatment history of the jars and the fixation of the samples. As sometimes samples were fixed in formaldehyde and later transferred to ethanol, we measured the formaldehyde concentration in every selected jar by a simple calorimetric test as described in Schiller et al. [18] (Table 1).

**Table 1:** List of taxa and material analysed

Class	Species	Number NHMW	Collection year	Formol [mg/l]	PCR success
Bivalves	*Dreissena polymorpha* (Pallas, 1771) [16]				
		19505	1891	0	18
		19513	1891	0	33
		19538	1892	0	48
		55324	1924	40–60	0
		248	1950	0	15
		84428	1985	0	80
		101710	1991	0	70
		101711	1991	0	65
		101850	1991	0	78
		101721	1991	0	70
		89907	1994	0	68
		89905	1994	0	75
		89906	1994	0	60
		83846	1994	0	85
		83847	1994	0	78
		89911	1994	0	80
	*Mimachlamys varia* (Linné, 1758) [2]				
		21777	1894	0	6
		87212	1969	20–40	19
Cephalopods	*Sepia plangon* Gray, 1894 [2]				
		55206	1884	0	13
		15467	1884	0	25
Gastropods	Stylommatophora				
	*Aegopis verticillus* (Lamarck, 1822) [2]				
		25272	1897	0	13
		73992	1912	0–10	44
	*Arianta arbustorum* (Linné, 1758) [3]				
		89915	1989	0	94
		86813	1991	0	94
		100810	1992	0	100
	*Arion subfuscus* (Draparnaud, 1805) [3]				
		31599	1885	0	0
		31600	1886	0	0
		100806	1999	0	50
	*Cepaea nemoralis* (Linné, 1758) [10]				
		19158	1892	0	18
		19185	1892	0	65
		23361	1895	≫100	8
		23362	1895	0	53
		25276	1897	0	28
		77921	1972	0	48
		74401	1973	0	73
		79088	1973	0–10	63
		87076	1974	0	40
		16017	1889 (+)	0	43
	*Deroceras reticulatum* (Müller, 1774) [2]				
		31593	1886	0	6
		45139	1908	0	0
	*Helix pomatia* Linné, 1758 [3]				
		74149	1891	0	50
		74402	1973	0	69
		101290	1993	0	56
	*Limax cinereoniger* Wolf, 1803 [2]				
		33545	1900	0	25
		77517	1926	0	31
	*Limax maximus* Linné, 1758 [3]				
		77545	1877	0	6
		31576	1885	0	0
		31574	1886	0	0
	*Malacolimax tenellus* (Müller, 1774) [2]				
		31587	1885	0	19
		31588	1885	0	6
Optisthobranchia	*Aplysia dactylomela* Rang, 1828 [3]				
		21012	1958	0	0
		73532	1955 (+)	0	6
		73533	1955 (+)	0	6
Basommatophora	*Lymnaea stagnalis* (Linné, 1758) [2]				
		86667	1987	0	63
		101939	1997	0	31
	*Planorbis planorbis* (Linné, 1758) [3]				
		84060	1985	0	75
		86681	1990	0	44
		86703	1991	0	31
Caenogastropoda	*Lyncina carneola* (Linné, 1758) [2]				
		37238	1897	0	38
		37237	1897	0	0
	*Viviparus acerosus* (Bourguignat, 1862) [6]				
		72362	1877	0	0
		77197	1969	0	20
		77201	1969	0	58
		102198	1992	≫100	5
		102197	1992	≫100	3
		101984	1995	0	10
	*Viviparus contectus* (Millet, 1813) [2]				
		101694	1991	10	19
		51317	1918 (+)	0	8
Neritimorpha	*Nerita orbignyana* Récluz, 1841 [2]				
		37296	1897	0	31
		37298	1897	0	25
Polyplacophorans	*Acanthopleura brevispinosa* (Sowerby, 1840) [2]				
		37333	1895	0	69
		37334	1895	0	100

Each line corresponds to one jar

Inventory number (NHMW), formaldehyde concentration in mg/l, and collecting date are given. (+) the given year is the time of determination (when the collection date is unknown)

Two individuals per jar were analysed; the total number of jars per species is given in parentheses besides the species names followed by the percentage of overall PCR success (all marker sequences; 1968 PCRs in total)

### DNA extraction

All lab work was performed in a DNA clean room and all utensils were sterilised and UV radiated before usage. For DNA extraction, two small pieces of tissue were cut off from the peripheral body region of each specimen and air dried to remove remaining ethanol. Each tissue sample was then treated with two different extraction methods: The first one is the low-cost Gen-ial First, All-tissue DNA-Kit (Troisdorf, Germany; in the following “Gen-ial-ATK”), which is based on several precipitation steps, without the use of toxic substances or columns. It was employed following the manufacturer’s protocol for small forensic material. This extraction kit was chosen because it is well established and regularly used for mollusc extractions in our lab. The second method, the Promega, Tissue and Hair Extraction Kit for use with DNA IQ (in the following “Promega-THK”; Madison, Wisconsin, USA), is based on magnetic beads, i.e., paramagnetic particles that bind DNA by their silica-coated surface. Magnetic separation of the beads allows quick purification steps without any columns or precipitation. It was chosen because it is especially dedicated for samples with expected low DNA concentrations. In both extraction methods lysis of cells was done by lysis buffer including Proteinase K. Extractions were performed following mostly the manufacturer’s standard protocols with two modifications. The first is the time of incubation in lysis buffer, which was extended in both cases to overnight (~12 h). The second is the volume of elution buffer in which DNA was finally eluted (40 µl). In all reactions negative controls were performed to screen for contaminated reagents: (1) control extractions without sample and (2) PCRs with distilled water instead of template DNA.

### PCR amplification and primers

All samples were tested for PCR success by amplifying two mitochondrial (mt) markers: To test larger amplicon sizes, we used the barcode region of the *cytochrome c oxidase subunit I* gene (COI) with primers that were designed for land gastropods in an earlier study [9]. These primers (COIfolmerfwd/COI_schneckrev) amplify a 700 bp sequence (COI-lf). Since our lab is currently performing a DNA barcoding project on molluscs (ABOL—Austrian Barcode of Life, pilot project Molluscs; http://www.abol.ac.at) covering a broad taxon sample, we could confirm the applicability of the COI-lf for all gastropod taxa. In addition, a shorter mt section was used to test amplification of DNA of lower quality: A partial region of the mt 16S rRNA gene, which we amplified with primers previously applied in a wide range of taxa (e.g. [9–11, 19, 20]) (16S_sch_fwd/16S_sch_rev; amplicon size ~400 bp; 16S-sf). Furthermore, for each of the species *C. nemoralis, D. polymorpha* and *V. acerosus/contectus*, specific primers for three short (overlapping) sections covering the mt COI barcoding sequence were used: LCO1490/Int1_nem, Int2f_nem/Int2r_nem, Int3f_nem/HCOvar; LCO1490/Int_1drei, Int2f_drei/Int2r_drei, Int3f_drei/HCOvar; LCO1490/Viv_int1R, Viv_int2F/Viv_int2R, Viv_int3F/HCOvar; amplicon size for all sections ~230 bp; COI-sf-1/2/3). All primer sequences are listed in Table 2.

**Table 2:** List of primer sequences, annealing temperatures (T_ann_ in °C) and target taxa

Primer name	Primer sequence (5′-3′)	Taxa used	T_ann_	Ref.
COIfolmerfwd	GGTCAACAATCATAAAGATATTGG	All taxa	56	[9]
COIschneckrev	TATACTTCTGGATGACCAAAAAATCA	All taxa	56	[9]
16S_sch_fwd	CGCAGTACTCTGACTGTGC	All taxa	50	[19]
16S_sch_rev	CGCCGGTCTGAACTCAGATC	All taxa	50	[9]
LCO1490	GGTCAACAAATCATAAAGATATT	*Cep., Drei., Viv.*	43	[21]
Int1_nem	GCTCAGATTGTTCATCCGAGG	*Cepaea*	43	TvR^a^
Int_1drei	YCTTACATTATTWARACGAGG	*Dreissena*	43	TvR^a^
Viv_int1R	AAATGCTATATCAGGAGCGCC	*Viviparus*	43	TvR^a^
Int2f_nem	GGTTCCACTGCTTGTAGGAGC	*Cepaea*	43	TvR^a^
Int2r_nem	CCCCAAAATTGAAGAAACACCCG	*Cepaea*	43	TvR^a^
Int2f_drei	GGTYCCRATAATACTTAAGTCTT	*Dreissena*	43	TvR^a^
Int2r_drei	GGCACGTATATTWCCTCATGTYC	*Dreissena*	43	TvR^a^
Viv_int2F	ATTGGTGGGTTTGGTAATTGGC	*Viviparus*	43	TvR^a^
Viv_int2R	ATAGAAGAAGCCCCAGCTAAATGCA	*Viviparus*	43	TvR^a^
Int3f_nem	GCGTCCGTTGACTTGGCCATT	*Cepaea*	43	TvR^a^
Int3f_drei	AGCTTCTTCWATTATRGCTTC	*Dreissena*	43	TvR^a^
Viv_int3F	GGCTCATGCTGGAGGTTCTGTAGA	*Viviparus*	43	TvR^a^
HCOvar	TAWACTTCTGGGTGKCCAAARAAT	*Cep., Drei., Viv.*	43	[21]

TvR^a^ = provided by TvR, unpublished data; W, Y–indicate wobble positions; *Cep* = *Cepaea*; *Drei* = *Dreissena*; *Viv* = *Viviparus*

During processing, DNA was stored at 4 °C and transferred to −20 °C for long-term storage. After 1 year, all samples were analysed again by amplifying the common DNA barcode region *COI* as well as the *16S* region (universal primers). This was done to test for potential quality loss of the extracted DNA over time. Finally, the DNA-concentration of all samples was measured with a biophotometer (D30, Eppendorf, Germany; see Additional file 1). The 260/280 nm ratio was in most cases below 1.8 indicating that there were no protein, phenol or other contaminants that absorb strongly at or near 280 nm.

The PCR conditions and polymerases were tested at the beginning of the project for all taxa and not changed afterwards. Subsequently, PCR for all *COI* amplifications was performed with the Phusion-Kit (High-Fidelity DNA Polymerase; Finnzymes, Espoo, Finland), while for *16S* the TopTaq-Polymerase-Kit (Qiagen) was used. Reactions with the Phusion-Kit were performed in 12.5 µl with 8.875 µl A. dest., 2.5 µl 5× Phusion GC Buffer, 0,25 µl 10 mM dNTP’s, 0.125 µl of each Primer [50 pmol] and 0.125 µl of the Phusion Polymerase and 0.5 µl of DNA. Reactions with the TopTaq-Polymerase-Kit were performed in 12.5 µl with 7.7 µl A. dest., 2.5 µl of Q-Solution, 1.25 µl 10× Buffer, 0.25 µl 10 mM dNTP’s, 0.125 µl of each primer [50 pmol] and 0.05 µl TopTaq Polymerase and 0.5 µl DNA. All PCR experiments included a positive control. To allow a reasonable comparison, we followed a standardized procedure with only one PCR for each primer combination (e.g., no repetitions with varying DNA concentration).

**Fig. 1:**
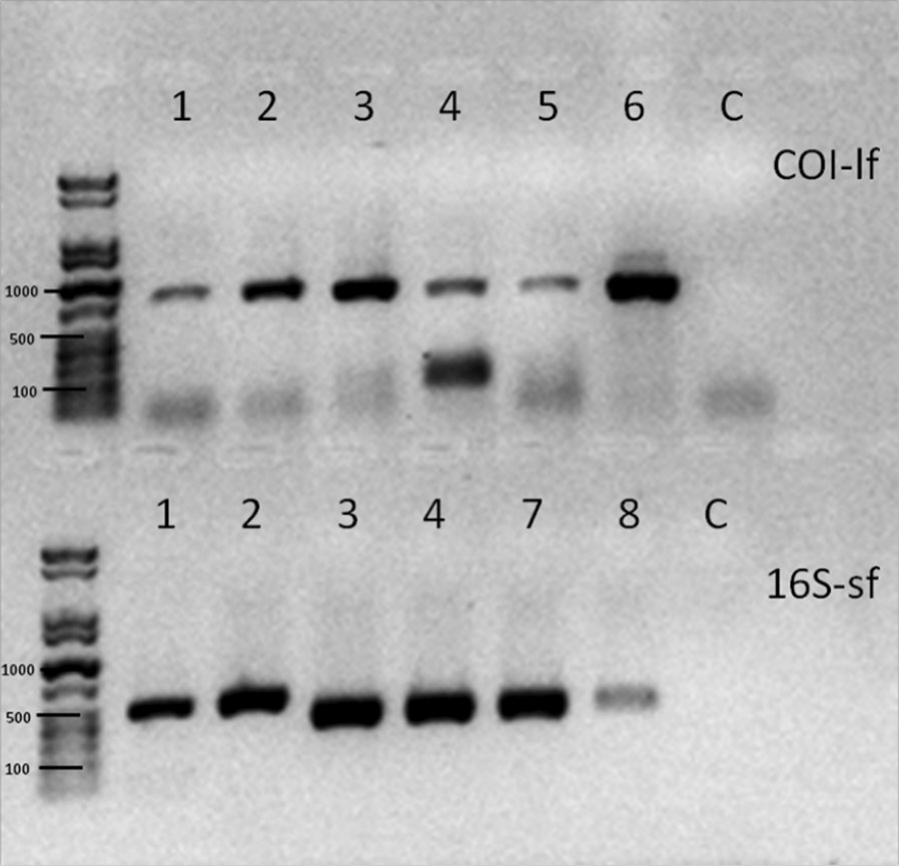
Representative image of an agarose gel electrophoresis for the amplicons COI-lf and 16S-sf (*1* = *A. brevispinosa* 37,334 A2, *2* = *A. brevispinosa* 37,334 A1, *3* = *A. arbustorum* 86,813 A2, *4* = *A. arbustorum* 100,810 A1, *5* = *D. polymorpha* 101,850, *6* = *D. polymorpha* 83,846 B1, *7* = *H. pomatia* 74,402 B2, *8* = *P. planorbis* 84,060 A2, *C* = Negative control)

The thermal cycling for the Phusion-Kit started with an initial phase of 98 °C for 30 s, followed by 40 cycles of 10 s at 98 °C, 30 s at annealing temperature, and 30 s at 72 °C. Final elongation was performed at 72 °C for 7 min. The thermal cycling conditions for TopTaq were as follows: initial phase at 94 °C for 4 min, then 35 cycles of 94 °C for 30 s, 30 s at annealing temperature, and 72 °C for 1 min. The final elongation lasted for 7 min at 72 °C. All samples were electrophoresed on 1 % (long amplicons) or 2 % (short amplicons) agarose gels. A representative gel image of COI-lf and 16S-sf of different taxa is shown in Fig. 1. To test whether the amplified amplicons are the expected gene sequences, a subset of samples was sequenced (LGC Genomics, Berlin) using the same primers used for producing the amplicons. Sequences were deposited in GenBank under the accession numbers KX537613-KX537636.

## Results

### Overall PCR amplification success

The results of the various PCRs of each individual are summarized in the Additional file 1. Although the results vary with respect to amplicon size and age and must be considered in detail, we first provide summarizing key findings (lumping the results of both extraction methods): Taking the results of all PCRs together (all primer combinations, 1968 PCRs in total), 43 % were successful. Concerning amplification of the COI-lf, 27 % were successful, while the shorter 16S amplicon (16S-sf) showed a significantly higher success rate (40.5 %). In the amplification of the overlapping COI amplicons (200 bp to 240 bp in length), which was performed in a subset of the taxa (68 individuals), 49 % yielded PCR products of the expected size, which is again significantly higher than with COI-lf. Detailed values for each of the primer pairs and for the two DNA extraction methods are provided in Tables 3 and 4. Concerning the measured DNA concentration, we found no correlation with either PCR success or sample age.

**Table 3:** Positive PCR results for all primer sets used [%] and comparison of the two extraction methods Promega-THK and Gen-ial-ATK (repetition of tests after 1 year not included)

Primer set	Promega-THK	Gen-ial-ATK
Total	38	48^a^
COI-sf-1 [215–230 bp]	50	53
COI-sf-2 [200–230 bp]	36	56^a^
COI-sf-3 [225–250 bp]	47	52
16S-sf [400 bp]	34	47^a^
COI-lf [700 bp]	24	30^a^

^a^Statistically significant, Chi square test; significance level p = 0.05

**Table 4:** Positive amplifications [%] in the first (year 1) and second (year 2) analysis for all samples in total, for the COI as well as for 16S sections for both extraction methods

		Year 1	Year 2
Total		34	32
Promega-THK	COI-lf	24	19
	16S-sf	34	42^a^
		29	31
Gen-ial-ATK	COI-lf	30	24
	16S-sf	47	42
		39	33^a^

^a^Statistically significant, Chi square test; significance level p = 0.05

For most taxa (15 out of 20) we verified authenticity of 16S-sf amplicons by sequencing (Additional file 2). In the other taxa the amount of amplification product was too low that sequencing failed. For the longer marker gene (COI-lf) sequences could be obtained only for 7 taxa, and for two taxa only short sections of the amplicons could be sequenced (Additional file 2).

**Fig. 2:**
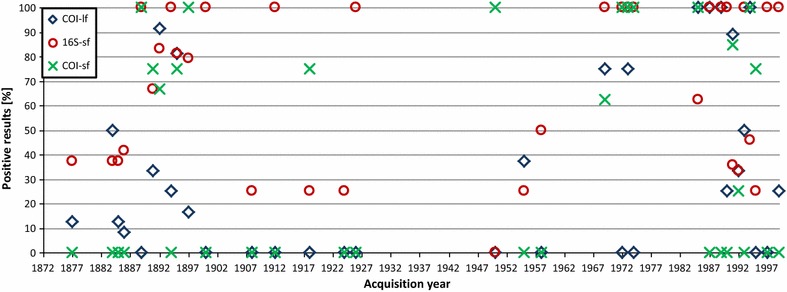
Positive results per year in percent for the three tested marker sequences (COI-lf, COI-sf, 16S-sf). *Symbols* on the 0 % *line* indicate samples that were completely negative

Of the two different extraction methods, Gen-ial-ATK performed significantly better. Throughout all taxa and with all primer sets, 48 % of PCRs were positive in samples extracted with Gen-ial-ATK, whereas 38 % were positive in samples extracted with Promega-THK (Table 3). This trend was valid in all detailed comparisons for the different primer sets separately, although only the results for 16S-sf and the COI-sf-2 are statistically significant (Table 3). Nonetheless, considering the results separately for various time spans (see below; Fig. 2), Gen-ial-ATK showed significantly better results in all tested primer sets in the time span 1877–1900 as well as 1985–1999, except for COI-lf. In the intermediate time period (1901–1985), Promega-THK performed better in all primer sets, although this is statistically not significant.

The repetition of the analysis after 1 year showed—with one exception—a slight decrease in the PCR success rate (e.g., in total for COI-lf and 16S-sf: 2 %). This decrease in PCR success occurred for both extraction methods as well as for large and small amplicons with the exception of 16S amplifications of samples extracted with the Promega-THK, where the number even increased (Table 4).

Formaldehyde traces did not inhibit amplification success in most cases, except at very high concentrations (over 60 mg/l). Although these observations are based on only a few samples in which formaldehyde could be detected at all (eight jars in total), they are noteworthy: None of the PCRs of samples from two (out of three) jars with formaldehyde concentrations ≫ 100 mg/l proved successful, while among the samples from the remaining six jars with low concentration traces of formaldehyde the success rate was 23 % (in 32 PCRs for each of the 48 DNA extractions). Overall, the 16S-sf worked significantly better than the COI-lf.

### Sample age

**Fig. 3:**
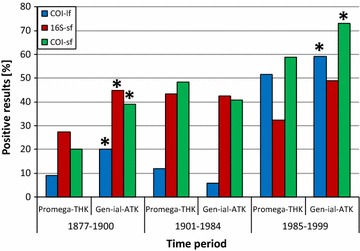
Comparison of PCR success in percent for the three different primer sets tested (COI-lf, 16S-sf, COI-sf) between the two extraction kits (Promega-THK/Gen-ial-ATK) and three time periods; *–statistically significant, Chi square test; significance level p = 0.05

PCR success is time-dependent concerning the age of specimens, but this is only a general trend. Our test set showed that many recent samples were negative, while even the oldest individuals provided positive results for the long and the short sections: *Limax maximus* from 1877 and *Sepia plangon* from 1884. Especially in the latter species the DNA concentration was high in some individuals (up to 20.6 ng/µl; see Additional file 1). In general, COI-lf showed a lower percentage of positive results in the older samples, especially for the early twentieth century, where no amplifications at all succeeded for this amplicon (Fig. 2). In contrast, amplification of the 16S-sf and the COI-sf amplicons in this time frame was successful in most of the cases. With one exception (1950), 16S-sf always gave positive results when COI-lf did not. All tested marker sequences in the more recent samples worked with higher percentages (between 30 and 70 %, see Fig. 3).

To further evaluate the results with respect to sample age, we split them into three time frames (Table 5): (1) 1877–1900 comprising the oldest samples up to 1900, when the use of formaldehyde fixation started; (2) 1985–1999 comprising younger samples up to 30 years old, (3) the intervening time frame (1901–1984) comprising medium- to old-aged samples with potential formaldehyde treatment. A comparatively longer time frame was selected for the middle period to compensate for the smaller number of individuals selected from the first half of the twentieth century. Overall, the results clearly show that the youngest samples worked best (48 %, n = 100), but that the samples from 1877 to 1900 also gave positive results (25 %, n = 120), similarly to the middle period 1901–1984 (26 %, n = 68) (Fig. 3). Interestingly, COI-lf worked poorly in the period 2 (1901–1984), even worse than in samples from period 1. No such clear difference among periods was observed in the success rate of 16S-sf and COI-sf (Fig. 3).

**Table 5:** Positive PCR amplifications [%] in total (Promega-THK & Gen-ial-ATK) for different time periods for all tested amplicon sizes

Positive results	1877–1900	1901–1984	1985–1999
Total	27	32	54
COI-lf [700 bp]	15	9	55
16S-sf [~400 bp]	36	43	41
COI-sf [~230 bp]	30	45	66

## Discussion

The present study comprehensively investigated mollusc specimens from the wet collection of the NHMW to test two DNA extraction methods in a broad variety of taxa using samples with widely differing samples ages. The significant difference in PCR success between the two extraction methods requires cautious interpretation and is discussed in detail below. Beyond that, our results confirm earlier investigations in several aspects: Even old specimens provide a good chance to isolate (albeit mostly short) DNA sequences, but a certain proportion of samples fail to yield positive results. Moreover, despite an effect of sample age—younger samples typically contain DNA of better quality—there is a high stochasticity in PCR success. This was demonstrated by repeating the PCR experiments after 1 year: PCR success is not completely reproducible. This general randomness is in agreement with observations during several of our phylogenetic studies based on museum samples (e.g. [22–25]) as well as with studies of other research groups [26–28]. In the following, we discuss the various factors that we tested and/or which could in our opinion influence the DNA quality and PCR success in mollusc specimens stored in alcohol.

### Sample age

The use of museum collections for barcoding analyses is a common topic recently [29, 30], and the chances of obtaining long PCR products is known to decrease with time because older DNA tends to be fragmented. Importantly, many recent studies have applied next-generation-sequencing approaches that require only short DNA amplicons. In such cases, sample age and DNA fragmentation are a lesser issue [31, 32]. For standard phylogenetic approaches, however, this problem is more evident and in some cases even the amplification of the standard DNA barcode region (corresponding to COI-lf in the present study) is not possible for older material. Our study also revealed an age effect: PCR success generally decreased with sample age. The correlation, however, was not linear. Each sample has an individual history that potentially influenced its DNA quality. Accordingly, age alone is a poor predictor of PCR success. This is clearly underscored by our results, in which many samples yielded positive results even with the whole barcoding region COI-lf (length ~700 bp). In a recent study on beetles and moths, Mitchell [33] proposed, even for samples that are older than 3 years, that it would be more efficient to amplify several small overlapping amplicons and combine them afterwards. The argument is that such an approach is advantageous because it starts with small sections that, from the beginning, would avoid a lot of trial and error. Note, however, that this “patchwork” approach bears certain pitfalls, especially in highly variable taxonomic groups Dealing with six primers instead of two, for example, raises the probability that one primer might be suboptimal. With both, the number of amplifications and the number of primers, the danger of amplifying contaminating DNA rises. Such contaminations may lead to chimeric sequences that might easily remain undetected unless the data are checked meticulously using phylogenetic comparisons.

Besides specimen age, the topic of DNA degradation during storage is rarely explored. Our second run after 1 year yielded partially incongruent results compared to the initial PCR experiments. In this study the PCR success was lower with the Gen-ial-Kit (minus 6 %) than with the Promega-Kit (minus 2 %), independent of amplicon length. One explanation is the stochasticity of PCR success with old DNA in general, but additional factors such as freezing and thawing, different elution buffers and other components of the kit used may play a role. The degradation of DNA during storage requires further investigation.

### Comparison of DNA extraction methods

As seen in the results above, the Gen-ial-ATK (48 % positive results) performed significantly better than the Promega-THK (38 % positive results; significance tested with Chi Square test, p = 0,05). Nevertheless, examining the results in detail shows that PCR success is to some extent random: in a considerable proportion of PCRs (~10 %), the Promega-THK extraction was positive while the Gen-ial-ATK negative or vice versa. This observation is especially crucial when comparing the results of the repetition after 1 year: some samples showed positive results with Promega-THK and negative ones with Gen-ial-ATK, although the year before they had been positive also with Gen-ial-ATK. Unfortunately, this random factor of PCR failures is hard to eliminate.

Comparing the two different extraction methods among the three different time spans, the Promega-THK performed better in all primer sets in the time interval 1901–1985. One could hypothesize that this is due to the specialisation of the Promega-THK to formalin-fixed material, as four of the jars from this time span contained formalin traces. This result should not be over-interpreted because the performance differences are actually very small and not significant. In general, our results suggest that the type of DNA extraction kit does not make a big difference concerning the success of PCR with DNA of mollusc samples. Similar results were found in other studies with different extraction kits/methods to ours. Skujiene and Soroka [3] tested three DNA extraction methods in fresh material of slugs (shell-less gastropods), one of them a commercial standard kit and the other two based on phenol/chloroform extraction or salt precipitation, respectively. Their results showed that the ready-to-use kit generally performed better. Similar results were obtained by Popa et al. [8], who tested three commercial kits and a phenol/chloroform/isoamyl alcohol protocol. The commercial kits performed equally well, while efficiency was low using the phenol/chloroform/isoamyl alcohol protocol even though the latter method yielded the highest DNA concentration. The authors interpreted this as reflecting possible traces of remaining phenol that inhibited PCRs. Unfortunately, these studies are difficult to compare because they involved different methods and sample ages. The multitude of available kits further complicates final conclusions. Anyway, the extraction kit should be selected based on the age and preservation of the samples, although experience and handling are doubtless also important factors, especially for sensitive material such as old museum samples. It therefore seems reasonable to start with a kit well-established in the laboratory and modify the protocol, e.g., prolong the incubation time in the lysis buffer.

### PCR success and primer specificity

Among all mollusc taxa investigated, gastropod samples worked best, especially the land snails. While amongst the marine molluscs the polyplacophoran taxon *Acanthopleura* showed positive results in 80 % of all reactions, the analysed samples of marine bivalves and gastropods as well as cephalopods poorly worked with both tested primer sets. In some cases the results seem to reflect a problem with primer specificity, which could be expected in an analysis ranging over a major phylum such as the molluscs. For example in the bivalve *Dreissena*, we achieved good results with all target gene sections tested (52 % positive in total), except for 16S-sf (9 % positives). The amplification of the 16S-sf turned out to be problematic also for *Mimachlamys varia*, the other bivalve taxon included in this study. The fact that COI-lf worked well in these taxa could be a hint that the 16-sf primer set does not bind well in these mussels. *Cepaea*, which yielded in only 44 % positive reactions, was especially unsuccessful with COI-sf-3 (HCOvar/Int3f_nem) and COI-lf. In this case we know from previous studies [11] that the primer set for the long barcoding region amplifies well in this genus; we therefore assume the poor results are due to the age effect: more than half of the *Cepaea* samples were collected before 1900.

These results reflect an intrinsic problem in studies of this sort, i.e. testing extraction methods as well as effects of sample age and sample conservation on a broad taxon sample. The use of “universal” primers has certain drawbacks because the primer binding efficiency (primability and stability) is probably not identical in all species analysed. To ameliorate these effects, we used in addition more specific primers in some of the taxa. Confirmation of primer binding by testing positive controls (fresh samples) is not always the solution because even slightly reduced primer binding (not apparent when working with good-quality DNA) might perform worse with degraded DNA of low concentration. The fact that in some taxa amplicons were of such low concentration that sequencing failed, could also be due to primer binding problems in these cases. Such potential primer binding problems are compounded by taxon-specific problems. The slugs (shell-less gastropods; in this study representatives of the genera *Aplysia, Arion, Deroceras, Limax, Malacolimax*), for example, turned out to yield mostly poor results. Thus, our study confirmed the presumed problem of DNA analyses in mucopolysaccharide-rich mollusc taxa [2–5, 7]. Interestingly, the two extraction methods performed equally well in the slug taxa, whereas over the whole taxon sample, Gen-ial-ATK performed significantly better than Promega-THK.

### Fixation, conservation

The results presented here confirm our experience with other taxonomic groups [22, 24, 25] that the maximum amplicon length varies individually and does not strictly correspond with a specimen’s age, but also depends on the respective collectors and on curatorial aspects (the process comprising fixation, sample conservation and long-term storage). Among these factors, formaldehyde traces are especially relevant. In our study, formaldehyde traces (10–60 mg/l) in the jars did not have a major effect on PCR success. Nonetheless, the results show a clear difference according to amplicon size: the shorter the tested section, the higher the success rate despite formaldehyde preservation. Many studies tested various methods of DNA extraction from formaldehyde-preserved material, and several new methods were proposed (e.g. [34, 35]). Our study did not reveal any significant differences in success between the two tested extraction methods, confirming other studies involving tests of extraction-kits for formaldehyde-fixed material [35–37].

At the NHMW the use of formaldehyde for conservation started in 1896 [18]. It probably first became standard procedure in the early twentieth century, which agrees with our measurements. Especially in the 1901–1984 period, for which we obtained many negative results, nearly 1/3 of the jars contained formaldehyde traces. The results reflect both issues with formaldehyde and degraded DNA, thus due to DNA–DNA-crosslinks and fragmentation caused by formalin, only short sections may have been amplifiable. Furthermore, problems in DNA amplification are probably caused by bad DNA conservation in general. Nonetheless, we were able to amplify the short amplicons in several of these samples, and only COI-lf gave fewer positive results. The potential DNA amplification problem due to formaldehyde was probably compounded by bad DNA conservation in general. Many different extraction methods have been developed for mucopolysaccharide-rich taxa because mucopolysaccharides are thought to inhibit proteins such as proteinase and thus make DNA extraction difficult [6]. The present study showed a clear correlation between PCR success and mucopolysaccharide richness in various taxa. The slugs, for example, yielded mostly poor results. Our interpretation of this finding is that the problem lies not in the extraction itself, but in the first fixation phase. Our experience shows that slugs lose abundant water and slime during the fixation process and that the ethanol must be changed every other day, sometimes over several weeks. If this is not done properly, then the alcohol will become diluted and the mucopolysaccharides will inhibit fixation and accelerate DNA degradation. This interpretation is supported by our experience in other projects, where fresh and properly preserved slug samples yielded positive results. Another piece of supporting evidence is that, in the present study, the shorter amplicons could be amplified in several slug samples, whereas amplification of COI-lf was negative.

Finally, the fixation of some mollusc taxa is problematic due to their morphology. Thus, *Viviparus* showed the worst results (only 14 % positive reactions). None of the five primer sets tested worked well in this species. Viviparids have an operculum to close the aperture, and this apparently seals the shell very well. During the killing process they immediately close their aperture. This hinders the entry of fixation fluid, leading to an uncontrolled fixation in which the DNA degrades rapidly. A good way to preserve such molluscs is to insert a toothpick between shell and operculum, preventing complete closure and enabling fixation fluid to enter easily and quickly.

## Conclusion

In summary, DNA extraction from alcohol-preserved, mucopolysaccharide-rich taxa was quite successful with both extraction methods, although one of them, Gen-ial-ATK, performed significantly better. In general, PCR success decreases with sample age, but several other factors (taxon, age, fixation, conservation, extraction, primer specificity) might also influence the results in various ways. Since the key factors of fixation and sample storage are often poorly documented, PCR success in DNA from museum specimens will always have an element of unpredictability and stochasticity. To optimize the chances of success, several recommendations can be given based on our results and previously published data: (1) Primers should bind perfectly, and thus, primer selection/design should be done carefully. Suboptimal primers might amplify well in high quality DNA of fresh material, but fail to yield good results in degraded, low concentration DNA of old museum samples. When the sample quality is low—due to formaldehyde fixation, high age, imperfect conservation, etc.—longer amplicons are also more difficult to amplify. Ideally, primer sets for the desired sequences as well as for overlapping partial sequences should be at hand and used when PCR of longer amplicons fails. (2) Although in our study the PCR success was significantly better with one of the two extractions methods, using a kit already established in the lab is a good start because individual experience in routine laboratory procedures is also an important factor. The performance difference of the DNA extraction methods tested here is quite small, and other extraction methods (not tested in the present study) might perform equally well. When establishing a new DNA extraction method dedicated to museum samples in a lab, kits designed for forensic material and expected low DNA concentrations are very promising; as suggested by earlier studies (e.g. [3]) traditional phenol chloroform extractions might be suboptimal. (3) There are several potential reasons why some taxa performed unsatisfactorily (slugs, *Viviparus*, *Aplysia*), the most important being degraded DNA of poorly conserved specimens due to operculum-sealed shells or high mucopolysaccharide content. (4) All these aspects should be considered when fixing and storing fresh specimens. Curators should optimize conservation by repeatedly changing the alcohol until fixation is completed and ensure that the alcohol intrudes into the tissue. Finally, (5) each sample has an individual history that potentially may have influenced its DNA quality. Yet, especially for older material, information on maintenance of collections and specific preservation conditions over time is in many cases missing. Anyhow, maintaining the preserving conditions in the collection over time is essential.

## Additional files

10.1186/s13104-016-2147-7 In the additional file a detailed list of all samples used in this study can be found, including sample age and the inventory numbers. Furthermore, the DNA concentration [ng/µl] for both extraction methods (Promega-THK and the Gen-ial-ATK) and the formaldehyde content [mg/l] are given. The PCR success of all samples is indicated as well.
10.1186/s13104-016-2147-7 The additional file shows a table of all 20 taxa analysed in this study together with information of which species sequences of the COI-lf and the 16S-sf could be obtained.
